# Assessment of high-fat-diet-induced fatty liver in medaka

**DOI:** 10.1242/bio.031534

**Published:** 2018-11-15

**Authors:** Koichi Fujisawa, Taro Takami, Yumi Fukui, Takahiro Nagatomo, Issei Saeki, Toshihiko Matsumoto, Isao Hidaka, Naoki Yamamoto, Takeshi Okamoto, Makoto Furutani-Seiki, Isao Sakaida

**Affiliations:** 1 Center for Regenerative Medicine, Yamaguchi University School of Medicine, 1-1-1 Minami-Kogushi, Ube Yamaguchi 755-8505, Japan; 2 Department of Gastroenterology and Hepatology, Yamaguchi University Graduate School of Medicine, 1-1-1 Minami-Kogushi, Ube, Yamaguchi 755-8505, Japan; 3 Systems Biochemistry in Pathology and Regeneration, Yamaguchi University Graduate School of Medicine, 1-1-1 Minami-Kogushi, Ube, Yamaguchi 755-8505, Japan

**Keywords:** Medaka, Liver, Fatty liver, Ultrasound, Metabolomics

## Abstract

Fatty liver, which has been continuously becoming more common in a number of patients, is the most common liver disease. For detailed analysis, a useful model for fatty liver is needed and fish are considered as a potential candidate. We assessed through direct observation of the liver, which is the most conventional method for non-invasive analysis of progression in fatty liver. By using transparent medaka (*Oryzias latipes*), we were able to observe changes in fat deposition in the liver. An analysis of the progression of fatty liver using ultrasound showed a significant increase in echo intensity, which indicates that this is a useful examination method. In addition, we clarified a metabolite profile in the medaka liver fed a high-fat diet (HFD), which had not previously been shown in detail. This medaka model, allowing non-invasive and repetitive assessment, is a useful model for the analysis of diseases that cause fatty liver in which changes in detailed metabolites are identified.

## Abstract (precis)

**Summary:** Our medaka model allows for non-invasive and repetitive assessment and is useful in the analysis of fatty liver in which changes in detailed metabolites are identified.

## INTRODUCTION

Lifestyle-related diseases such as fatty liver, dyslipidemia, diabetes and hypertension are closely associated with unbalanced diet, lack of physical activity and excessive stress. Because of their association with obesity or insulin resistance, they have become a major health issue in modern society. In particular, fatty liver disease (also called hepatic steatosis), which is a general term for hepatic disorders caused by triglyceride deposition in hepatocytes due to over-nutrition, is increasingly prevalent and has become the most common hepatic disease. The type of hepatic steatosis that occurs in patients who drink little or no alcohol is called nonalcoholic fatty liver disease (NAFLD) and can be further divided into simple fatty liver – which has a favorable prognosis – and progressive nonalcoholic steatohepatitis (NASH) – which has a possibility of progressing into cirrhosis/liver cancer (Lapadat et al., 2017). Although mice models have been used in studies aimed at the development of treatments inhibiting this progression, a new, more efficient model is desired (Asgharpour et al., 2016).

Small fish, including medaka (*Oryzias latipes*) and zebrafish (*Danio rerio*), have attracted particular attention as new model organisms (Goessling and Sadler, 2015; Matsumoto et al., 2010). Assessing the process of hepatic steatosis with a minimally invasive method is important to obtain stable results. The simplest way is to directly observe changes of the liver, but this is difficult to achieve non-invasively in most organisms. However, mutants of medaka have been reported in which the body color is light, allowing the direct observation of introduced cells and organs such as the heart and liver (Antinucci and Hindges, 2016). On the other hand, methods that provide more detailed information on the changes taking place in the liver, such as ultrasound imaging, are considered useful for the assessment of fatty liver. Although the use of ultrasound imaging for the characterization of liver cancer progression (Goessling et al., 2007) and the evaluation of heart function (Ernens et al., 2016) in zebrafish has been demonstrated, no study exists for assessing fatty liver in medaka using this method.

In this study, we used optical observation and ultrasound imaging to non-invasively monitor the progression of high-fat diet (HFD)-induced hepatic steatosis in transparent medaka (Shima and Shimada, 1991; Shimada et al., 2005). In addition, we evaluated a metabolite profile of the liver in medaka fed an HFD.

## RESULTS

### Optical assessment of hepatic steatosis

**Fig. 1.**
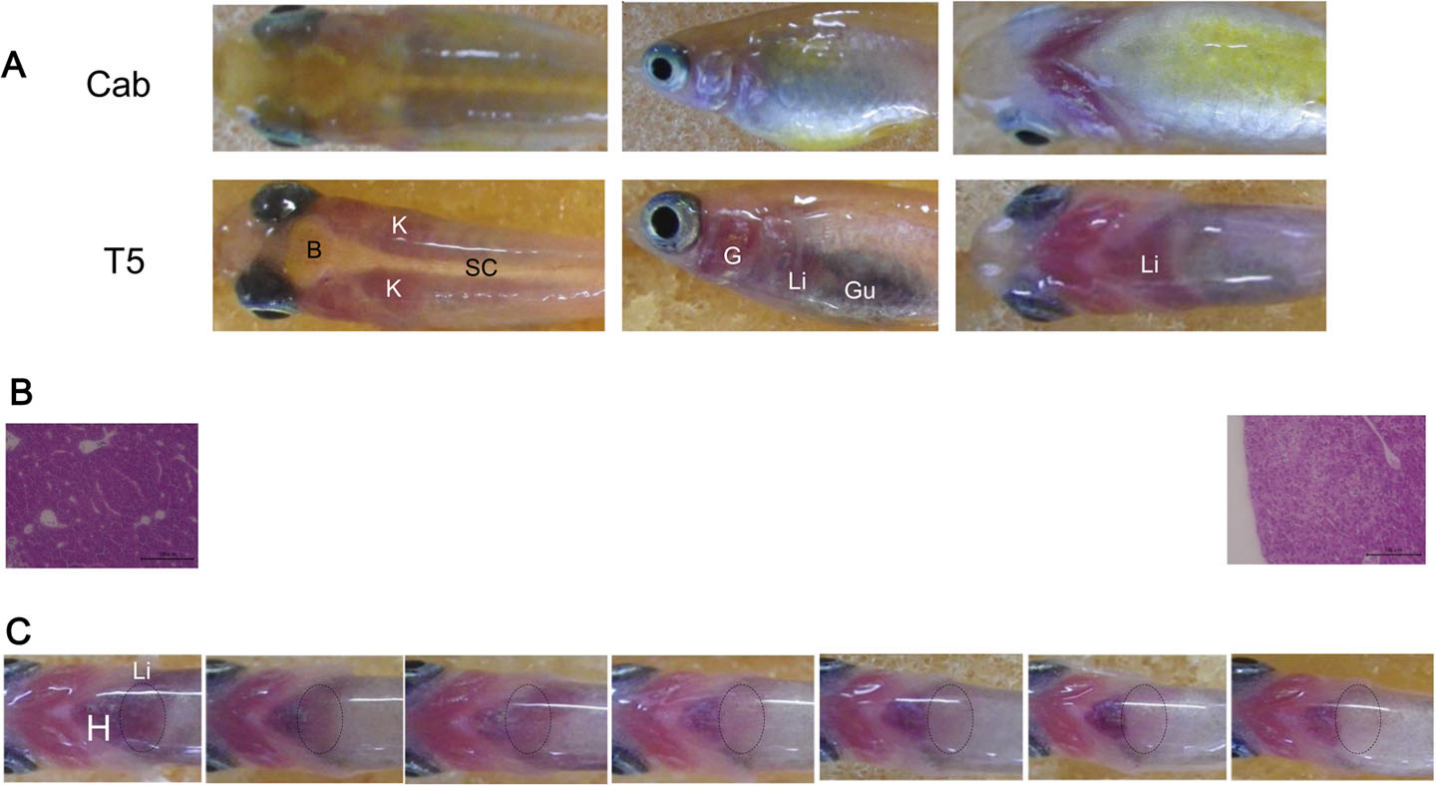
**Non-invasive optical assessment of fatty liver progression.** (A) Comparison between transparent and wild-type medaka. Top, wild-type medaka (cab); bottom, transparent medaka (T5); left, ventral abdominal view; center, lateral abdominal view; right, dorsal cephalic view. (B) HE staining of liver sections. Left, liver prior to HFD feeding; right, liver after 12 weeks of HFD. (C) Macroscopic changes in transparent medaka due to HFD feeding. The liver is encircled by a dotted line (*n*=8). B, brain; K, kidney; SC, spinal cord; G, gill; Li, liver; Gu, gut; H, heart.

Wild-type medaka, such as the Cab strain which is generally used in research, does not allow visual observation of internal organs from outside the body. However, there are pigmentation mutants in the fish whose bodies are transparent. One of these is the T5 strain, which was described by Shimada and Shima (2004). As seen in Fig. 1A, the liver in the T5 strain is visible from outside of the body, in contrast to the wild-type Cab strain. We subjected individuals of the T5 strain to an HFD in order to optically evaluate the progress of steatosis. Photographs were taken every 2 weeks up to week 12. The heart remained a red color, while the liver gradually turned to a white color, a change attributed to fat deposition. Hematoxylin-Eosin (HE) staining at week 12 confirmed a marked fat deposition (Fig. 1B,C).

### Assessment of hepatic steatosis by ultrasound imaging

**Fig. 2.**
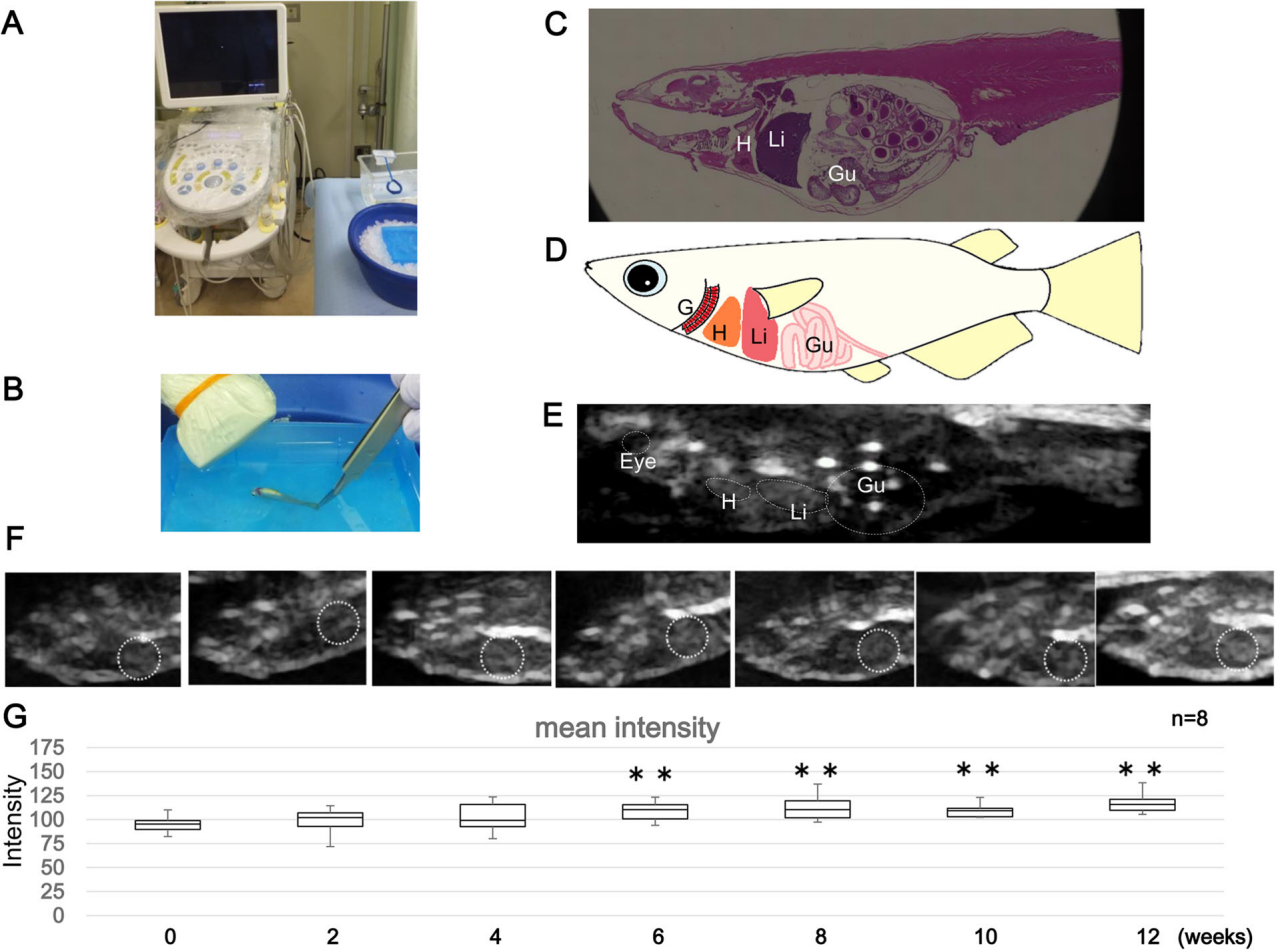
**Non-invasive assessment of fatty liver progression by** **ultrasound.** (A) Ultrasound equipment used. (B) Ultrasound scanning using an ultrasound linear probe. (C) HE-stained image of a sagittal section in adult medaka. (D) Drawing showing the positions and shapes of various organs in the medaka body. (E) Ultrasound image of the whole medaka body. The positions of specific organs are indicated by dotted lines. (F) Assessment of fatty liver progression by ultrasound imaging in HFD-fed medaka. The liver is encircled with a dotted line. (G) Changes in echo intensity due to fatty liver progression (mean intensity) (*n*=8, Student's *t*-tests,*P<0.05; **P<0.01). G, gill; H, heart; Li, liver; Gu, gut.

Parallel to optical observation, we assessed the progression of fatty liver in more detail using ultrasound imaging. The used equipment is displayed in Fig. 2A. Before the scan, animals were immobilized by immersion in cold water containing tricaine (Fig. 2B). The eyes, heart, liver and intestine were successfully imaged and identified (Fig. 1C-E). The analysis of changes taking place in the liver revealed an increasing echo intensity, which indicated growing steatosis (Fig. 2F). Histogram analysis showed that the mean intensity values at week 8 to 12 were statistically significantly higher (Fig. 2G).

### Changes in metabolites due to HFD feeding

**Fig. 3.**
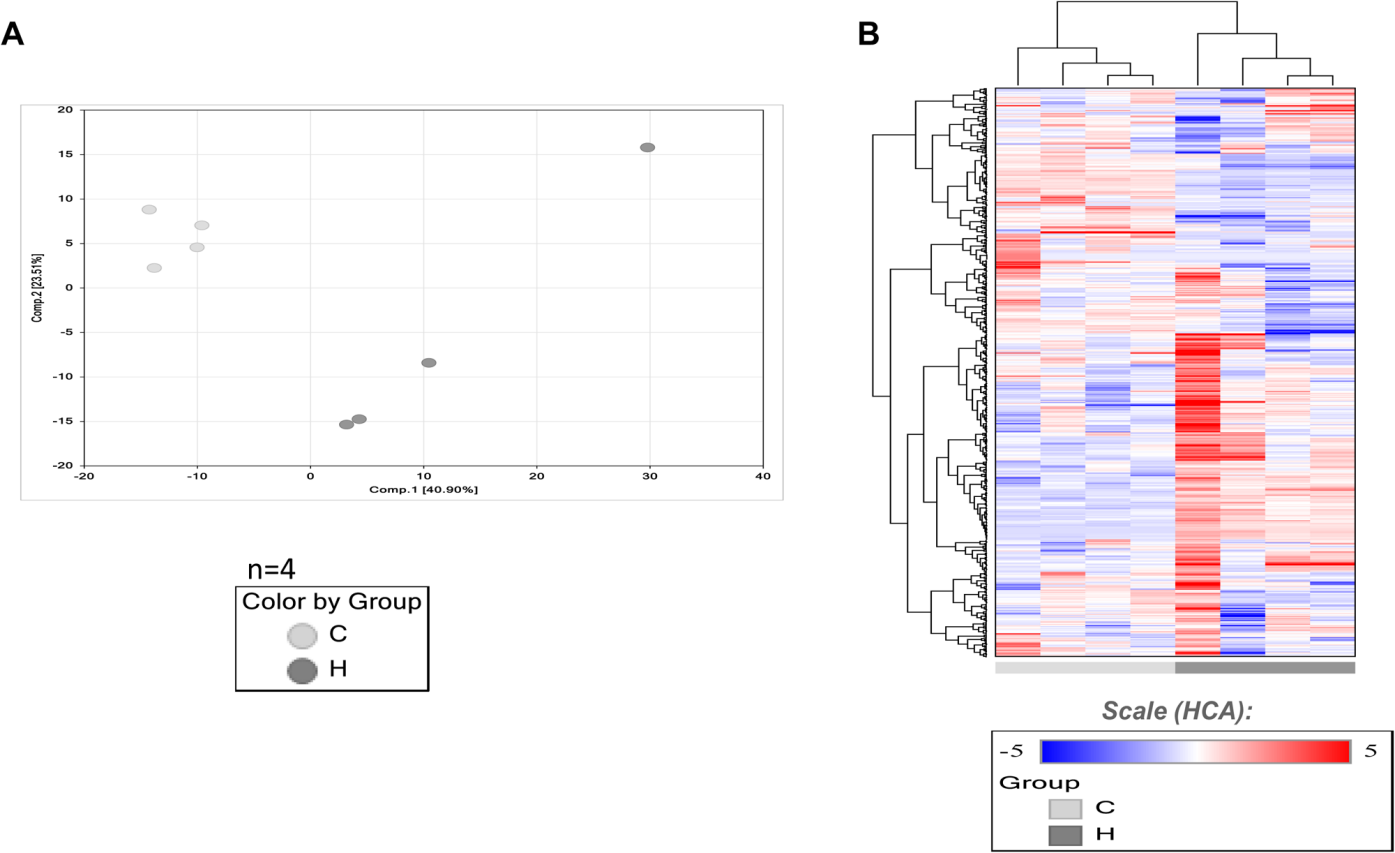
**Metabolome analysis and serial analysis of gene expression (SAGE).** (A) PCA of normalized metabolic data derived from liver samples of medaka fed an HFD for 2 months (*n*=4) and control group (*n*=4). Percentage values indicated on the axes represent the contribution rate of the first (PC1) and second (PC2) principal components to the total amount of variation. (B) Heat map of the hierarchical cluster analysis. The columns indicate the HFD and the control groups. The rows indicate the normalized levels of each metabolite. The dendrogram for each heat map shows the relation of the normalized metabolite level patterns.

The above analyses demonstrates the usefulness of HFD-fed medaka in the assessment of fatty liver. Although understanding the states of detailed metabolites is important, no detailed study has been reported. Therefore, we examined changes in metabolites due to HFD feeding in this study. We compared changes in metabolites using the liver samples isolated from the Cab medaka fed an HFD for 8 weeks and the control Cab medaka fed a normal diet by metabolome analysis. Principal component analysis (PCA) showed that the HFD-fed group and the control group were clearly separated on the X-axis (Fig. 3). Ingenuity Pathways Analysis (IPA) demonstrated increases in metabolites suggesting the involvement in the lipid metabolism including concentration of lipid, synthesis of lipid and accumulation of lipid and hepatic inflammation, including release of reactive oxygen species and entry into S-phase of hepatocytes (Table 1). Concerning changes in metabolites, for long-chain saturated fatty acids, increases in myristoleate (14:1n5) and oleate/vaccenate (18:1) were observed. As for unsaturated fatty acids, decreases in omega-3 unsaturated fatty acids and increases in omega-6 unsaturated fatty acids were observed (Table 2). In addition, there were increases in metabolites associated with phosphatidylcholine (PC), phosphatidylethanolamine (PE), phosphatydylinositol, diacylglycerol and sphingolipid (Table 3) and in those associated with glycolysis: pentose metabolism, glutathione and amino acids (Table 4).

**Table 1.**
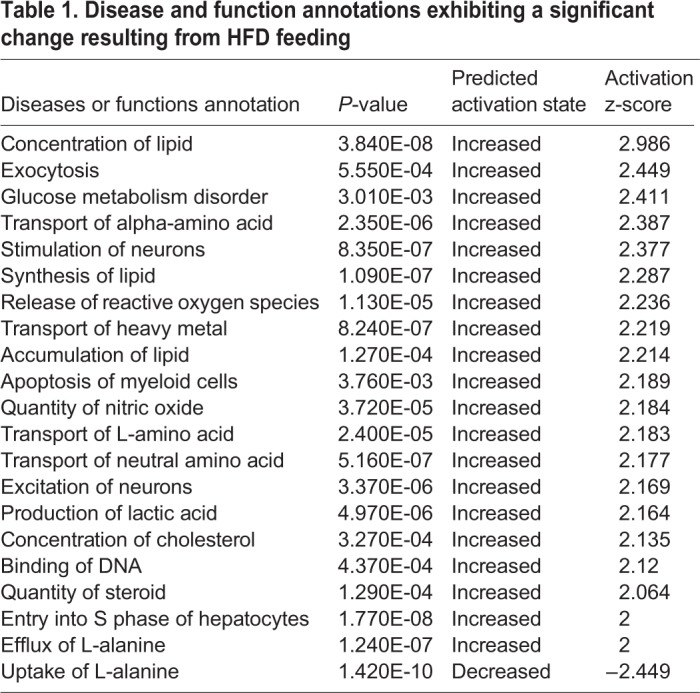
**Disease and function annotations exhibiting a significant change resulting from HFD feeding**

**Table 2.**
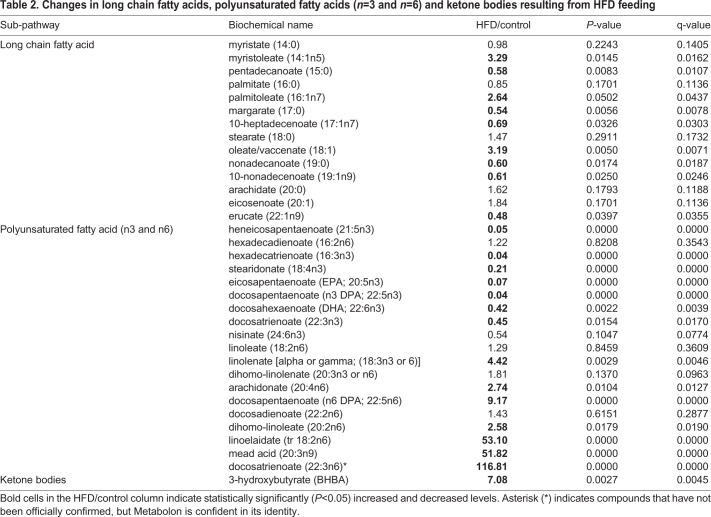
**Changes in long chain fatty acids, polyunsaturated fatty acids (*n*=3 and *n*=6) and ketone bodies resulting from HFD feeding**

**Table 3.**
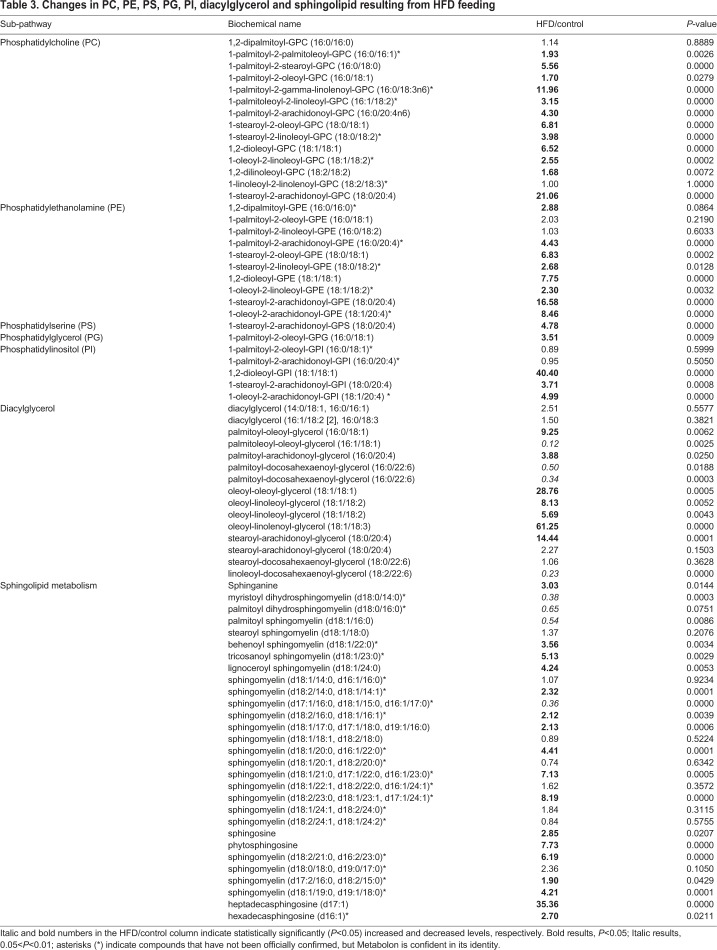
**Changes in PC, PE, PS, PG, PI, diacylglycerol and sphingolipid resulting from HFD feeding**

**Table 4.**
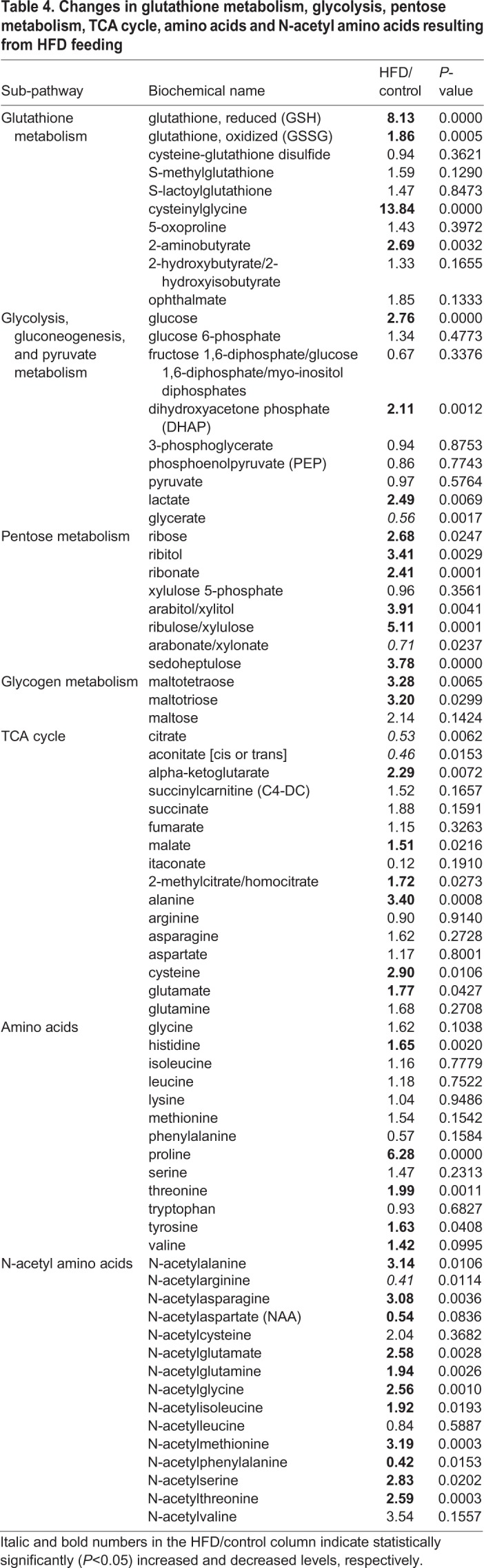
**Changes in glutathione metabolism, glycolysis, pentose metabolism, TCA cycle, amino acids and N-acetyl amino acids resulting from HFD feeding**

## DISCUSSION

Fatty liver disease is highly prevalent, may progress to cirrhosis or liver cancer and increases the risks of various lifestyle related diseases. Therefore, new models for analyzing the detailed mechanisms of the disease and testing novel therapies are required. We have previously reported the usefulness of medaka as a model of hepatic steatosis (Fujisawa et al., 2017). In the present study, we performed a more detailed analysis of changes in metabolites accompanying hepatic steatosis and assessed non-invasive methods for monitoring fatty liver progress in this model.

Although the direct observation of the liver from outside the body would be an ideal non-invasive method for the assessment of fatty liver, this is difficult in most organisms, including wild-type medaka. Thus, we employed the T5 strain, which have transparent bodies, allowing relatively easy viewing of organs, such as the heart, from outside the body (Shima and Shimada, 1991; Shimada et al., 2005). In the present study, we were able to observe a gradual liver opacification and an increase in the abdominal adipose tissue in HFD-fed T5 medaka. However, as several strains of transparent medaka with different transparency traits have been reported (Iwamatsu et al., 2003; Wakamatsu et al., 2001), future studies should examine which model is the most appropriate for the observation of the liver.

As a more quantitative method, we performed ultrasound imaging. The ultrasound findings showed that this method allows repetitive sequential observation of the abdomen in the same medaka. Moreover, the combination of ultrasound imaging with direct observation gives a more detailed assessment of fatty liver progression.

Increases in echo levels in the liver parenchyma, hepatorenal contrast, vascular blurring and deep echo attenuation are typical findings in patients with fatty liver disease (Idilman et al., 2016). In the present study, a marked elevation in echo levels was observed, but we could not successfully assess vascular blurring or deep echo attenuation. In addition, organs with a small change in fat deposition that corresponds to hepatorenal contrast need to be identified to advance assessment. Future development of higher-performance ultrasound probes is expected to allow for more detailed analysis (Huang et al., 2017). Recent studies reported the use of magnetic resonance imaging (MRI) (Ueno et al., 2016) and computed tomography (CT) (Seo et al., 2015) for assessing fatty liver in medaka and zebrafish, respectively. Therefore, further studies on the usefulness of the combination of the aforementioned methods are necessary.

We performed a detailed analysis of the metabolome changes taking place in the liver of medaka fed an HFD, providing important information on the metabolic pathways associated with fatty acids, phospholipids, glutathione metabolism and energy metabolism in this model organism (Shin et al., 2014). The HFD increased lipid metabolites in medaka liver, which was also shown in previous reports on HFD-fed mice (Kim et al., 2011). In addition, an increased level of glucose was also reported in HFD-fed mice (Patel et al., 2017). Concerning anti-oxidative reaction, enhanced glutathione (GSH) biosynthesis caused by partially reversed energy and lipid metabolism disturbance was observed in HFD-fed rats (Song et al., 2013).

Concerning changes in long-chain saturated fatty acids, there was an increase in oleate/vaccenate (18:1), which corresponds to the fact that HFD contains 64.9% oleic acid, 12.8% palmitic acid (C16:0), 7.6% stearic acid (C18:0), 10.3% linoleic acid and 0.2% α-linolenic acid (Matsumoto et al., 2010). However, palmitic acid and stearic acid levels in medaka did not significantly increase, despite their levels being high in HFD. Therefore, to understand complex lipid metabolism pathways, detailed analysis using labeled compounds is desirable. Corresponding to the presence of a high amount of linoleic acid and a low amount of α-linolenic acid in an HFD, decreases in omega-3 unsaturated fatty acids and increases in omega-6 unsaturated fatty acids were observed, suggesting that our model is more prone to developing inflammation, as changes in the ratio of these fatty acids are known to result in an alteration in anti-inflammatory activity (Lazic et al., 2014). In addition both reduced and oxidized forms of glutathione, which are involved in antioxidant effects, increased. There was also an increase in L-gamma-glutamylcysteine, a compound in the synthetic pathway, which suggests that the necessity of antioxidative effects by glutathione increases due to the HFD. Remarkable increases in PC, PE, phosphatydylinositol, diacylglycerol and sphingolipid in the HFD-fed group are considered to be a result of the metabolism from compounds in an HFD taken to major components of the cell membrane: lipids. In particular, lipid droplet, which is ubiquitously present not only in adipocytes but also in hepatocytes, is a mass of neutral fats surrounded by a single layer, composed mainly of triglycerides and sterol esters, increases as hepatic steatosis progresses. Therefore, synthesized lipids are considered to be used as components of lipid droplet.

To sum up, this study demonstrated the ability to non-invasively and repeatedly assess hepatic steatosis in transparent medaka through optical observation and ultrasound diagnostic equipment, suggesting its potential as a model for fatty liver research.

## MATERIALS AND METHODS

### Ethics statement

All fish were maintained and used in experiments in accordance with the Animal Care Guidelines of Yamaguchi University. All animal studies have been approved by Yamaguchi University, approval number is 21-038.

### Experimental model

Two different medaka (*O. latipes*) strains were used. The inbred medaka strain (Kyoto-Cab) was used in this study. Six-month-old female himedaka strain Cab (an orange-red variety of medaka, *O. latipes*) fish were used for the HFD steatosis analysis and the metabolome analysis. Transparent medaka (T5 strain), kindly provided by Dr. Shima (Shima and Shimada, 1991; Shimada et al., 2005), were used in the experiments where the progress of fatty liver was assessed (approximately 6-month-old females).

### Diets

The protein, fat and carbohydrate content, as well as the fatty acid compositions, of the control diet and the HFD were described in a previous report (Matsumoto et al., 2010). The energy content of the control diet (Hikari Crest; Kyorin Co. Ltd, Hyogo, Japan) was 3.3 kcal/g, with 25.3% of the calories from fat, 62.5% from protein and 13.8% from carbohydrates. The energy content of the HFD (HFD32; CLEA Japan Inc., Tokyo, Japan) was 5.1 kcal/g, with 56.7% of calories from fat, 20.1% from protein and 23.2% from carbohydrates.

### Ultrasound imaging

We used an HI VISION Ascendus ultrasound diagnostic apparatus and an EUP-L52 linear probe (central frequency: 5.5 MHz) (Hitachi Ltd., Tokyo, Japan). Six-month-old female medaka (T5) were fed an HFD. At each time point (weeks 0, 2, 4, 6, 8, 10 and 12), after first optically observing the change in the color of the liver from outside the body, the intensity of the liver was measured by placing a probe in medaka anesthetized in water containing tricaine (*n*=8). Changes in the intensity values of the liver were calculated and were assessed with mean intensity values as changes of the group.

### Metabolome analysis

Metabolomic and statistical analyses were conducted at Metabolon as described previously (Shin et al., 2014). Briefly, cell pellets were subjected to methanol extraction and then split into aliquots for analysis by ultrahigh performance liquid chromatography/mass spectrometry (UHPLC/MS) in the positive, negative or polar ion mode, as well as by gas chromatography/mass spectrometry (GC/MS). Metabolites were identified by automated comparison of ion features to a reference library of chemical standards followed by visual inspection for quality control.

### Statistical analysis

To determine statistical significance in ultrasound analysis, Student's *t*-tests were used, with *P*<0.05 considered significant. To determine statistical significance in metabolome analysis, Welsh's two-factor *t*-tests were performed using Array Studio (Omicsoft) or ‘R’ to compare protein-normalized data between experimental groups, with *P*<0.05 considered significant.
